# Self-administered complementary and alternative methods of treating mental disorders among students in Wrocław: a cross-sectional study

**DOI:** 10.3389/fpubh.2025.1734137

**Published:** 2026-01-08

**Authors:** Jakub Sobieraj, Jakub Sleziak, Michał Szyszka, Marta Błażejewska, Kamila Łukańko, Pola Soczomska, Kinga Bodziony, Patryk Piotrowski

**Affiliations:** 1 Faculty of Medicine, Wrocław Medical University, Wrocław, Poland; 2 Departament of Psychiatry, Wrocław Medical University, Wrocław, Poland

**Keywords:** alternative medicine, ashwagandha, CAM, depression, marijuana, Poland, substance use, university

## Abstract

**Introduction:**

Mental health disorders such as depression are a rising issue among university students. Some of them use complementary and alternative medicine (CAM) as self-administered therapy instead of or together with professional care. Defining the scale of the problem, its underlying reasons and possible implications are crucial for addressing it in clinical psychiatry and public health strategies.

**Methods:**

A cross-sectional survey on students from Wrocław universities was conducted between April 2024 and December 2024. The form developed specifically for this study contained questions about demographic status, respondents’ mental health history, satisfaction with psychiatric or psychological help and factors affecting it. Survey also assessed experience and attitudes towards various CAM methods, including non-pharmacological like exercise, meditation, yoga and pharmacological such as herbs, e.g., st. John’s wort (*Hypericum perforatum*), supplements, psychedelics, fly agaric (*Amanita muscaria*), marijuana and other non-conventional therapies. To evaluate current depressive symptoms, questions modelled on the Patient Health Questionnaire (PHQ-10) were used. 493 responses were included in the statistical analysis.

**Results:**

46.5% of respondents had a history of mental disorders, with depression being the most prevalent (74.7%). While 45.3% of all students reported consultations with a psychiatrist and 44.8% usage of antidepressants, 96.1% applied CAM, mainly: physical exercise (81.4%), meditation (60.5%), yoga (39.1%). From herbs, the most popular were *Melissa officinalis* (53.0%) and *Withania somnifera* (24.8%), and from other substances: marijuana (31.3%), vitamins (22.5%) and psychedelics (10.4%). The main obstacles we identified in obtaining professional care were cost (80.7%), availability (35.7%) and fear of stigma (30.7%). Acceptance for classic therapies varied from 81.2% for psychotherapy and 75.9% for psychiatric drugs to 16.2% for electroconvulsive therapy (ECT). The factors affecting propensity to use particular CAM modalities included sex and severity of disorder. Females preferred herbs, probiotics and vitamins, while more males reported intake of *A. muscaria*. We found that among those students who engaged in professional health care services, there was significantly higher usage of marijuana, vitamins and probiotics. What’s more, users of marijuana, ashwagandha and st. John’s wort presented more intense depressive symptoms, based on PHQ-10. In the case of marijuana, its use is more prevalent among yoga practitioners and students with attention deficit hyperactivity disorder (ADHD). Consumption of psychedelics, marijuana and meditating is also connected to higher acceptance for novel therapies, such as ketamine and psilocybin, and practising yoga—for ketamine alone.

**Conclusion:**

Prevalence of CAM use among students is high. One of the reasons we identified is limited access to professional psychiatric help. Use of CAM without supervision may potentially lead to adverse events. Psychiatrists treating students should consider those risks. Public health strategies should include educating students about CAM and classic therapies such as ECT, developing clinical guidelines for managing patients who use CAM and improving accessibility to mental health care for students. Future research should focus on studying the issue in other communities and populations, as well as precisely assessing the risks and potential benefits of particular CAM methods.

## 1 Introduction

Mental disorders are a growing threat to public health worldwide, with anxiety and depression being the most prevalent diagnoses. According to data from 2015, 280 million people worldwide suffer from depression, which accounts for 3.8% of the population. The WHO estimated that by 2021, this number had increased to 350 million, which constitutes 4.4% of the global population (1–3). Moreover, according to the Polish National Health Fund, in 2020 the number of days off due to mental disorders, adaptive disorders, and stress amounted to 17.6 million. These figures represented nearly a twofold increase compared to 2012 (4).

A population particularly vulnerable to psychiatric disorders is young people, including university students. Studying at university can be associated with increased stress, especially among medical students and female students (5, 6). According to a study conducted in Poland by Karmolińska-Jagodzik et al., 23.5% of students suffer from mild depression, while 6.5% experience moderate or severe depression (7). Moreover, 33.9% regularly feel down and sad, and 28.98% noticed an increase in nervousness compared to the period before starting their studies. A new threat in the population of students is smartphone addiction, which can be associated with increased risk of depression (8, 9).

One of the major issues faced by people suffering from depression is limited access to professional psychiatric help. According to data published by the National Health Fund in February 2025, the average waiting time for admission to a mental health hospital for children is 266 days, while for adults it is 162 days (10). Conversely, due to the shortage of specialists, commercial consultations are a significant financial burden. In developing countries, there is a significant shortage of professionals, leading to long waiting periods and, in some cases, even to a lack of psychiatric care availability (11). In Poland, the situation is particularly alarming in the case of psychiatric care for children and adolescents. Problems such as a shortage of specialists and overcrowding of hospitals result in admissions of children to psychiatric units for adults (12).

Complementary and alternative medicine (CAM) is a term for medical practices and products that are not part of standard medical care, including such methods as meditation, yoga, dietary supplements, herbs, and massage (13). CAM for treatment of depression can improve its symptoms, however existing recommendations and guidelines utilising CAM in therapy vary in quality (14, 15). Evidence of CAM effectiveness from scientific research appears to be low in quality, and further studies are needed to assess their clinical usefulness and safety (16, 17). However, many CAM methods are presumably popular due to their high availability and low cost. A proper diet that provides the necessary nutrients in adequate amounts, such as omega-3 fatty acids, folic acid, and group B vitamins is crucial for maintaining general health, and dietary interventions can have a positive impact in the treatment of depression (18, 19). Regular physical activity can influence mental health by changes in neuroplasticity, inflammation, oxidative stress, the endocrine system, self-esteem, social support and self-efficacy (20), as well as biochemical balance in the central nervous system, including changes in activity of serotonin, brain-derived neurotrophic factor and glutamate (21–23). Another activity used in alternative approaches in depression treatment is yoga. While studies assessing its efficacy in alleviating depressive and anxiety symptoms are mainly low-quality evidence due to methodological drawbacks, yoga may be beneficial in improving mental health (24–26).

The use of self-administered dietary supplements and unprescribed or illegal drugs for treating depression is also a challenge for public health. Substances like marijuana or psilocybin, lysergic acid diethylamide (LSD) and other psychedelics used for self-treatment can bear negative consequences to health. The use of cannabinoids can negatively affect cognitive functions, particularly in younger individuals, impairing their academic performance, as well as increasing the risk of developing schizophrenia (27, 28). Threats to somatic health include higher risk of myocardial infarction and stroke (29, 30), respiratory system impairment (31) and adverse neonatal outcomes in women exposed to cannabinoids during pregnancy (32). Research shows promising results in the use of psychedelics in the therapy of psychiatric disorders such as depression and anxiety. Conversely, administration of psilocybin bears a risk of serious adverse events, such as worsening of depression, suicidal behaviour, psychosis, and convulsive episodes. More common are mild adverse events: headache, anxiety, nausea, fatigue, dizziness and elevated blood pressure (33–35). Studies also have shown that high doses of psilocybin caused extreme fear in 30% of participants (36). More research is needed to assess the risks of self-administration of psilocybin and LSD by psychiatric patients.

The aim of the study is to determine the prevalence of use of CAM methods for treating mental disorders among students at Wrocław universities, with a special focus on herbal preparations and psychoactive substances. The study also aims to identify factors influencing students’ choice of these methods, and to elucidate the mechanisms behind choosing self-administered therapies instead of seeking professional help. A better understanding of this subject can lead to the development of public health strategies and recommendations for primary care physicians and mental health specialists.

## 2 Methods

### 2.1 Study design and setting

This research is a cross-sectional survey study conducted between April 2024 and December 2024. Data were collected using an anonymous online questionnaire (Google Forms).

A multi-channel recruitment strategy was employed to maximise reach among students of Wrocław universities. The survey link was distributed through: (1) the project’s dedicated Facebook page “Psychiatria też dla ludzi” (Psychiatry for People Too), where promotional posts were regularly published; (2) Facebook groups specifically created for students of Wrocław Medical University; (3) personal social media accounts of the research team members, leveraging networks of Wrocław university students; (4) in-person promotion during lectures at Wrocław University of Science and Technology, where students were encouraged to participate; and (5) printed posters and flyers displayed at strategic locations within Wrocław Medical University, including the dean’s office, the university hospital where students attend clinical rotations, and the university library. Additional printed materials were placed at the University of Wrocław and Wrocław University of Science and Technology.

To ensure that only eligible participants completed the survey, the questionnaire clearly stated at the beginning that it was intended exclusively for students enrolled at Wrocław universities. Participants were required to indicate their study mode (full-time, part-time, or evening studies) and to specify their field of study by name. This initial section served both to verify eligibility and to collect key demographic information about the study sample.

The inclusion criteria were as follows: 18 to 40 years of age, students of public and private universities located in Wroclaw, students of all university degrees, including bachelor’s degree, master’s degree, doctoral degree, students of full-time and part-time university courses.

The exclusion criteria were: adult students of high schools, students from universities located outside of Wrocław, lack of answer to questions about age, sex, university, study mode and field, employment, and marital status.

### 2.2 Participants and sampling

The target population consisted of students currently enrolled in universities in Wrocław. Participants were eligible if they were actively studying at any Wrocław-based higher education institution at the time of the survey.

Informed consent was obtained electronically at the start of the questionnaire. Before accessing the survey questions, all participants were presented with an introductory statement outlining the study’s purpose, guaranteeing complete anonymity, clarifying the voluntary nature of participation, and informing them of their right to withdraw at any time without consequences. Proceeding beyond this introductory page constituted implied consent to participate.

Participation was fully voluntary and anonymous. Responses were stored securely in a password-protected digital environment accessible only to the research team.

A total of 499 responses were received between April 13, 2024, and December 6, 2024. Five responses were excluded due to incomplete data, and one was excluded because the participant was attending an adult high school rather than a university, resulting in a final sample of 493 students.

### 2.3 Survey instrument

The survey was developed specifically for this study and administered online via Google Forms. The questionnaire began with an introductory statement outlining the study’s purpose, guaranteeing response anonymity, and clarifying that participation was voluntary and could be terminated at any time without repercussions and that filling it implies consent to participate in the study. The instrument consisted of three main sections described.

#### 2.3.1 Sociodemographic data

The first section collected demographic information, including age, gender, study mode (full-time, part-time, or evening studies), field of study, employment status, and marital status.

#### 2.3.2 Mental health history and use of psychiatric care

This section assessed participants’ mental health history, including past psychiatric diagnoses and use of mental health services. Participants were asked about their satisfaction with psychiatric and psychological care and the factors that discouraged them from seeking professional help.

To evaluate depressive symptoms over a longer timeframe, we developed a modified questionnaire based on the Patient Health Questionnaire-9 (PHQ-9). The PHQ-9 is a well-validated screening tool corresponding to DSM criteria for major depressive disorder. We used the validated Polish version of the PHQ-9 (provided by Pfizer Inc., available at http://www.phqscreeners.com), which consists of nine core items scored from 0 to 3 (total range 0–27), plus an additional item assessing functional impairment. All original items and the scoring structure were retained, but we modified the timeframe from “over the past 2 weeks” to “over the past 12 months.” This modification was implemented to capture a broader temporal perspective on depressive symptomatology, which was deemed more relevant to the study’s objectives of understanding students’ mental health experiences and their use of complementary and alternative methods over an extended period. It is important to note that this instrument was not used for diagnostic purposes, but rather to screen for the presence and severity of mood disturbances in our study population.

#### 2.3.3 Use of complementary and alternative methods for mental health

The core of the survey assessed both conventional psychiatric interventions (such as psychotropic medications and psychotherapy) and various complementary or alternative approaches, including physical exercise, meditation, yoga, herbal supplements, dietary modifications, psychedelic substances, and other non-conventional methods. Participants were asked to rate their perceived effectiveness of these approaches.

Additional questions examined information sources for alternative therapies, whether participants consulted healthcare professionals about these approaches, and their willingness to recommend such methods to others.

### 2.4 Statistical analysis

Statistical analyses were conducted utilising the Microsoft® Excel (Version 16.89.1) data analysis ToolPack. Categorical data were expressed as counts and percentages, whereas quantitative data were represented as mean ± standard deviation (SD), median ± interquartile range (IQR), or 95% confidence interval (CI). The chi-square test was used to compare qualitative data between groups. Quantitative data analysis was performed using either the Student’s t-test or single-factor ANOVA, depending on the number of groups being compared. To analyse correlation relationships, Pearson’s correlation coefficient was calculated, followed by determining the t-statistic and converting it to *p*-values using Student’s t-distribution with n-2 degrees of freedom. A *p*-value of less than 0.05 was considered statistically significant.

## 3 Results

### 3.1 Sociodemographic characteristics of a sample

The sociodemographic characteristics of respondents, including age, sex, study mode, field of study, employment and marital status, as well as social media usage, cigarette smoking habit and previous psychiatric diagnoses is summarised in Table 1.

**Table 1:** Sociodemographic characteristics of the studied population.

Category/attribute	n (%)
Age	
Mean age	22.09
Youngest participant	18
Oldest participant	38
Sex	
Female	340 (69%)
Male	142 (28.8%)
Other	11 (2.2%)
Study mode	
Full time	442 (89.7%)
Part-time	44 (8.9%)
Evening	7 (1.4%)
Fields of study	
Health	247 (50.1%)
Humanities	42 (8.5%)
Engineering	112 (22.7%)
Social sciences	74 (15.0%)
Other	18 (3.7%)
Employment	
Working	267 (54.2%)
Not working	226 (45.8%)
Marital status	
Single	221 (44.8%)
Informal relationship	264 (53.5%)
Married	8 (1.6%)
Social media usage	
>3 h daily	229 (47.0%)
<3 h daily	239 (49.1%)
Several times in a week	17 (3.5%)
Once a week or less	2 (0.4%)
Smoking (tobacco)	
Yes	114 (23.1%)
No	379 (76.9%)
Mental health diagnosis history	
Yes	229 (46.5%)
No	264 (53.5%)
Including	
Depression	171 (74.7%)
Anxiety	148 (64.6%)
Eating disorders	52 (22.7%)
ADHD and ASD	49 (21.4%)
OCD	20 (8.7%)
Personality disorder	15 (6.6%)
PTSD	9 (3.9%)
Bipolar disorder	7 (3.1%)
Schizophrenia	1 (0.4%)

ADHD, attention deficit hyperactivity disorder; ASD, autism spectrum disorder; OCD, obsessive compulsive disorder; PTSD, post-traumatic stress disorder.

### 3.2 Experiences and attitude towards professional mental health care

**Figure 1:**
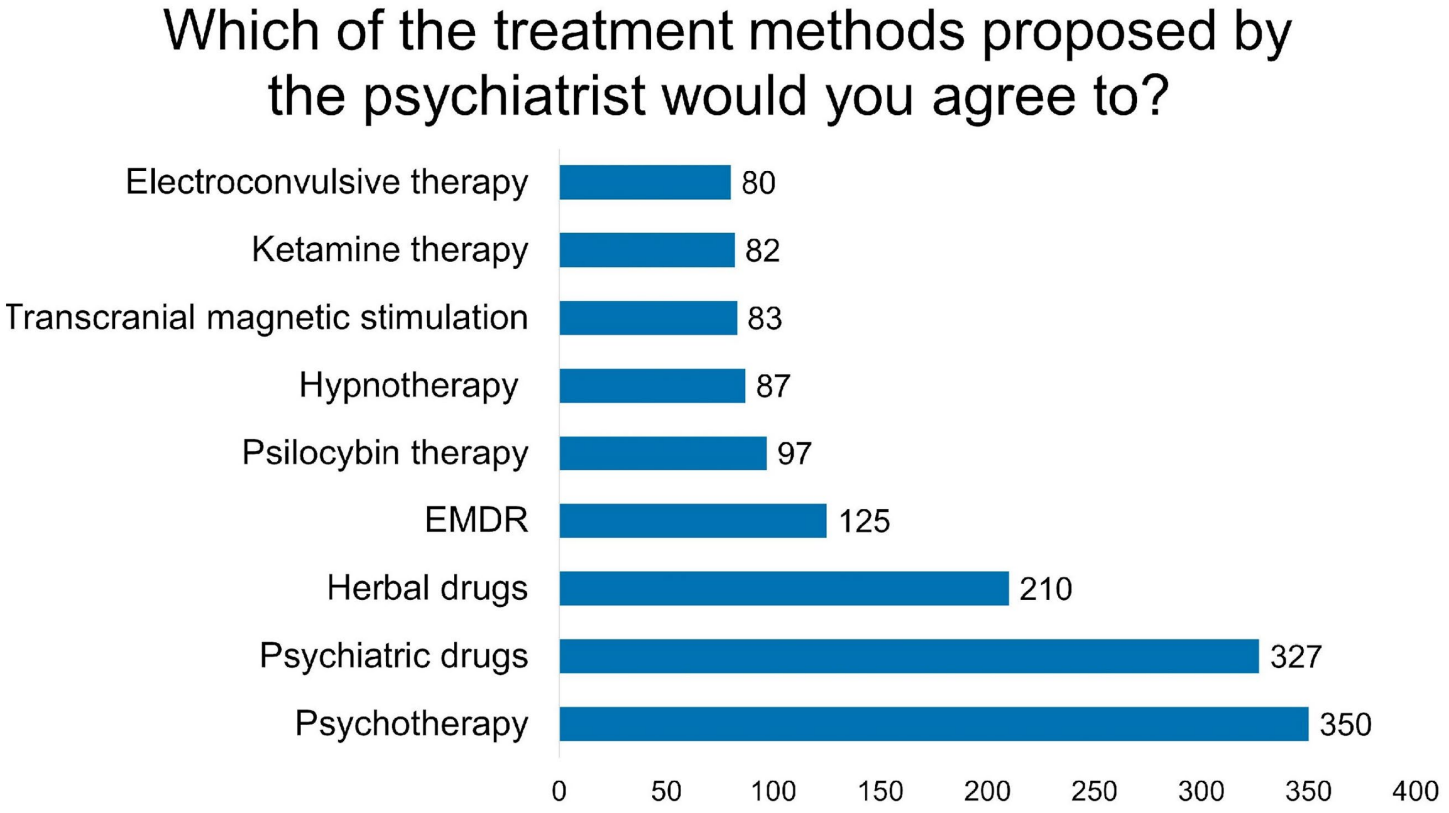
Acceptance of different psychiatric therapies.

Analysis of mental health service utilisation and satisfaction revealed varying patterns of engagement and acceptance across different treatment modalities (Table 2). Cost emerged as the primary barrier to psychiatric care access, cited by 276 (80.7%) participants, followed by limited service availability (122; 35.7%) and concerns about stigma (105; 30.7%). Additional obstacles included perceived lack of physician empathy (67; 19.6%), fear of judgment (37; 10.8%), and scepticism about treatment efficacy (34; 9.9%). Treatment acceptance patterns demonstrated the highest willingness for psychotherapy (350; 81.2%), followed by psychiatric medications (327; 75.9%). Analysis of psychological support services revealed that 135 (27.6%) participants were actively engaged in treatment. Regarding usage of all psychotropic drugs, 118 (24.3%) participants reported current use, 99 (20.4%) indicated past use, and 269 (55.3%) had never used such medications. Detailed utilisation patterns and satisfaction metrics are presented in Table 2 and Figure 1.

**Table 2:** Mental health service utilisation and patient satisfaction levels.

Service type	Utilisation pattern	*n* (%)	Satisfaction level	% of users
Psychiatric care	Ever consulted	223 (45.3)	Very high	30.5
			High	33.6
			Moderate	21.4
			Low	9.2
			Very low	5.3
Psychological support	Currently in treatment	135 (27.6)	Very high	28.7
	Previously utilised	219 (44.7)	High	38
	Never accessed	136 (27.8)	Moderate	17.9
			Low	7.2
			Very low	8.3
Psychotropic medications	Current use	118 (24.3)	Very high	33.9
	Past use	99 (20.4)	High	29.3
	Never used	269 (55.3)	Moderate	21.8
			Low	5.9
			Very low	9.2

The data represent participant experiences with mental health services. Percentages for utilisation patterns are calculated from total respondents; satisfaction percentages are calculated from those who had assessed each respective service on the scale 1–5 where 1 means “Very low” and 5 means “Very high”.

### 3.3 Utilisation of complementary and alternative medicine methods

**Figure 2:**
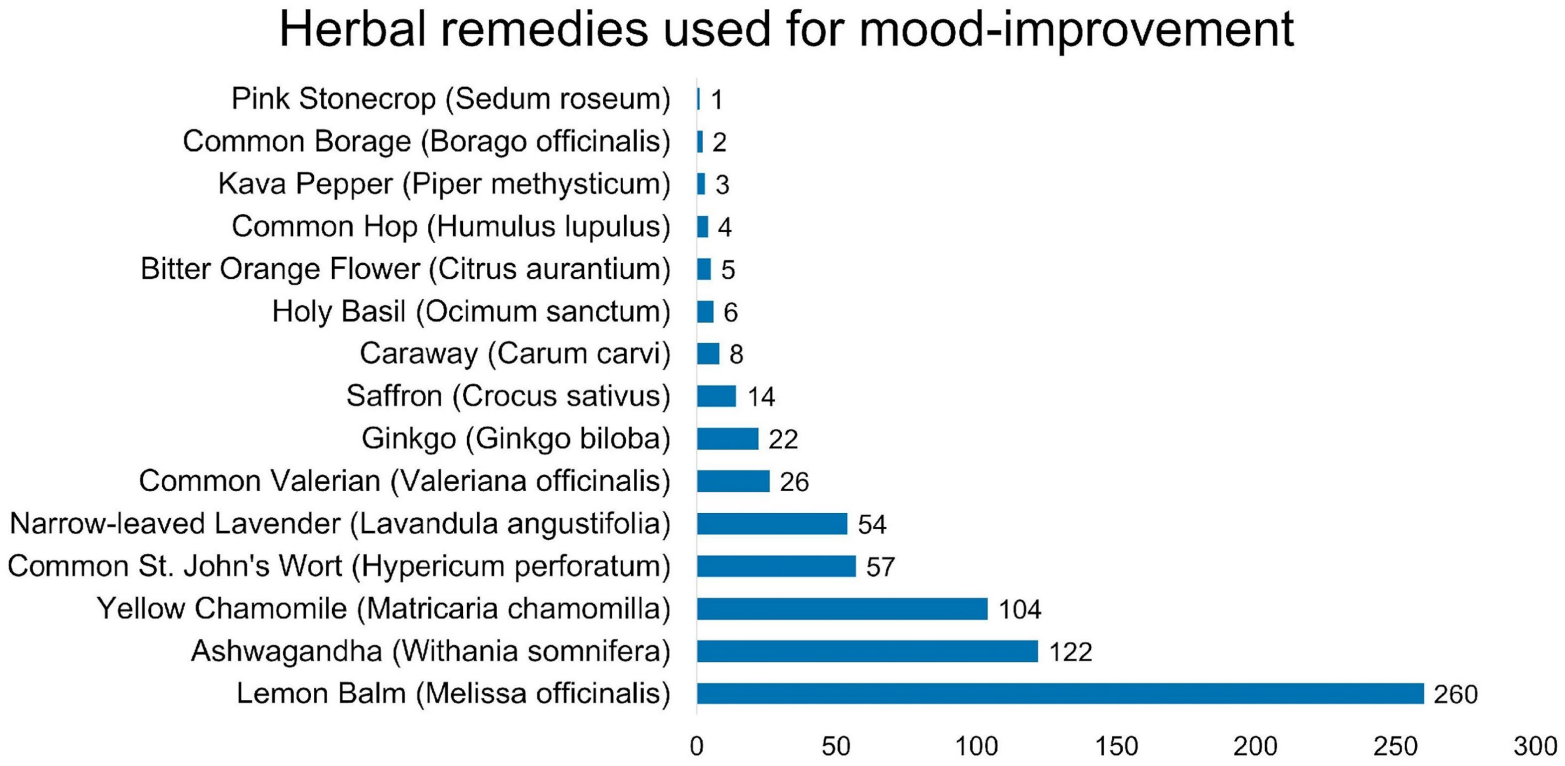
Prevalence of alternative methods for mental health improvement.

Analysis of alternative and complementary approaches to mental health care revealed diverse utilisation patterns and acceptance levels (Table 3). Among treatment modalities, herbal medicines demonstrated the highest acceptance. Self-initiated mood improvement strategies were widely adopted, with 474 (96.1%) participants reporting engagement in at least one method. Physical exercise emerged as the predominant strategy, followed by meditation practices and yoga. Satisfaction analysis among CAM users revealed predominantly positive experiences, with the majority reporting above-average satisfaction levels (ratings 4–5). Detailed information regarding acceptance of specific treatment modalities, utilisation frequencies of self-initiated strategies, and satisfaction distribution is presented in Table 3 and Figure 2.

**Table 3:** Acceptance and utilisation of complementary and alternative approaches to mental health care.

Category	Approach/Strategy	Acceptance/Utilisation *n* (%)
Alternative treatment modalities (acceptance among respondents, *n* = 431)	Herbal medicines	210 (48.7)
	EMDR therapy	125 (29.0)
	Psilocybin therapy	97 (22.5)
	Hypnotherapy	87 (20.2)
	Transcranial magnetic stimulation (TMS)	83 (19.3)
	Ketamine therapy	82 (19.0)
	Electroconvulsive therapy (ECT)	80 (18.6)
Self-initiated mood improvement strategies (utilisation among users, *n* = 474)	Physical exercise	386 (81.4)
	Meditation	287 (60.5)
	Yoga	193 (39.1)
	Dietary interventions	122 (25.7)
	Prayer	121 (25.5)
	Aromatherapy	71 (15.0)
	Hypnosis	44 (9.3)
	Holotropic breathing	32 (6.8)
	Acupuncture	10 (2.1)
Satisfaction with CAM for mood improvement (among CAM users, *n* = 442)	High satisfaction (rating 5)	83 (18.8)
	Very high satisfaction (rating 4)	155 (35.1)
	Moderate satisfaction (rating 3)	127 (28.7)
	Low satisfaction (rating 2)	51 (11.5)
	Very low satisfaction (rating 1)	26 (5.9)

The data represent acceptance of alternative treatment modalities (*n* = 431), utilisation of self-initiated strategies among those who reported using any such method (*n* = 474), and satisfaction levels among CAM users (*n* = 442). Percentages are calculated from respective denominators for each category. CAM, Complementary and Alternative Medicine; EMDR, Eye Movement Desensitisation and Reprocessing.

**Figure 3:**
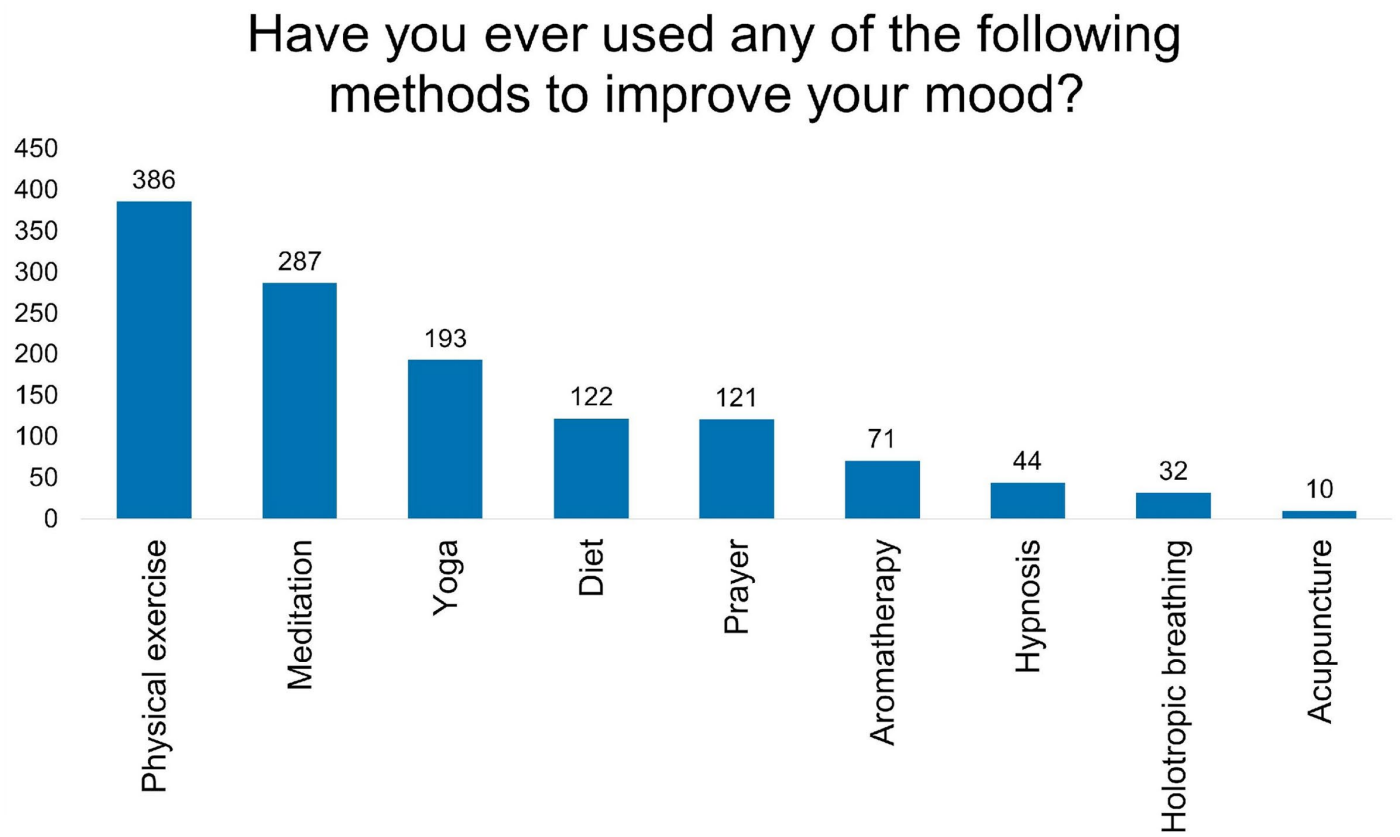
Prevalence of different herbs used among study respondents.

Investigation of herbal preparation usage (n = 491) revealed lemon balm (*Melissa officinalis*) as the most frequently utilised herb by 260 (53.0%) participants, followed by ashwagandha (*Withania somnifera*) at 122 (24.8%) and yellow chamomile (*Matricaria chamomilla*) at 104 (21.2%). Common st. John’s wort (*Hypericum perforatum*) and lavender (*Lavandula* sp.) showed moderate usage at 57 (11.6%) and 54 (11.0%), respectively. Notably, 179 (36.5%) participants reported no herbal supplement use. The prevalence of examined herbs usage was depicted in Figure 3.

**Figure 4:**
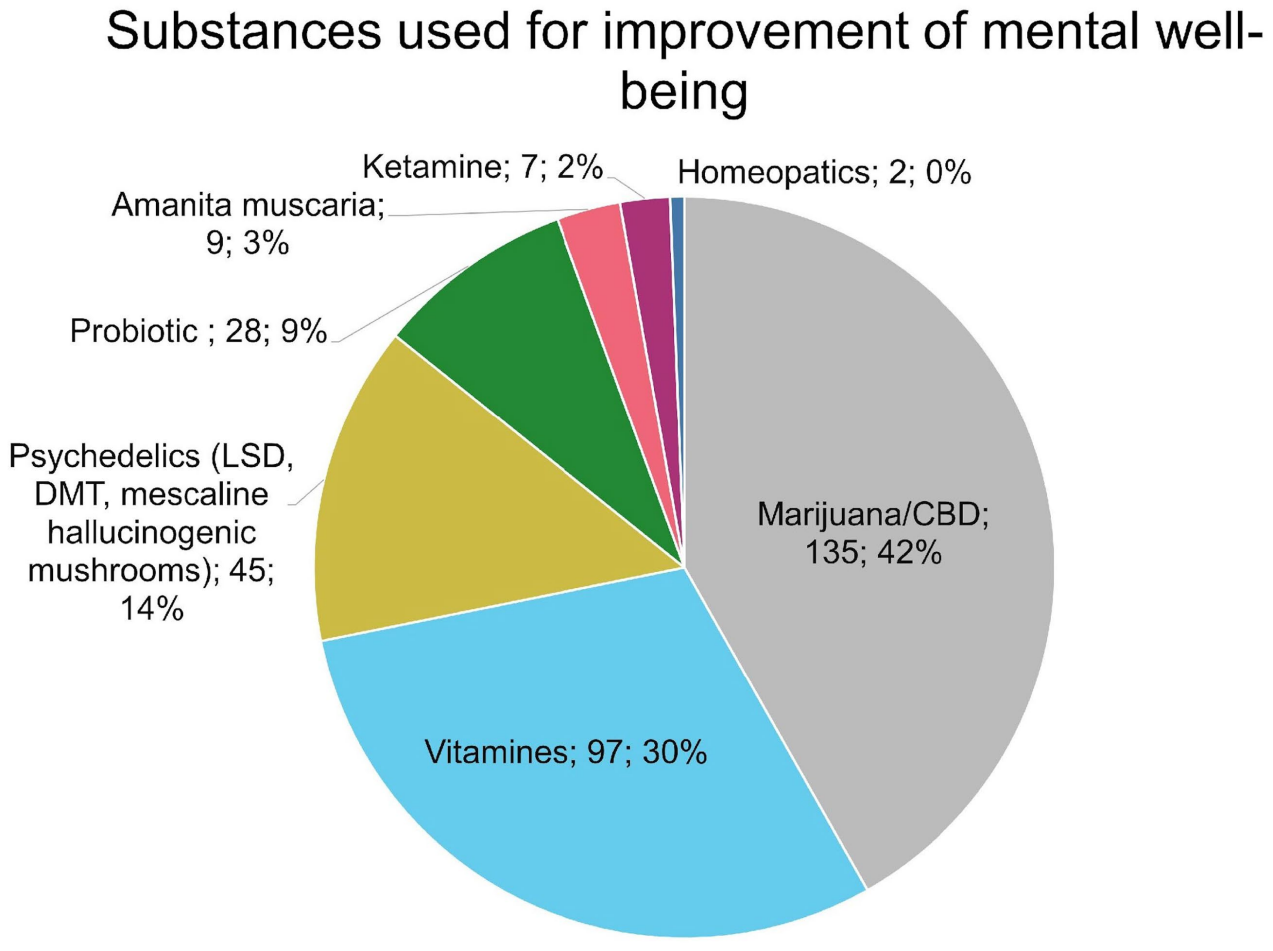
Number of participants using particular substances for the improvement of mental well-being.

Analysis of substance use for mental well-being (*n* = 432) showed that 233 (53.9%) participants reported no substance use. Among users, marijuana/CBD was most common (135; 67.8%), followed by vitamins (97; 48.7%) and psychedelics (45; 22.6%). The numbers of responders who reported usage of particular substances for improvement of mental well-being were depicted in Figure 4.

Satisfaction ratings for substance use (*n* = 270) indicated predominantly moderate effects, with 122 (45.2%) reporting moderate satisfaction, 60 (22.2%) high satisfaction, and 37 (13.7%) maximum satisfaction.

Information sources for alternative therapies (*n* = 367) were primarily media (288; 78.5%) and friends (201; 54.8%), with minimal consultation from medical professionals (86; 23.4%) or scientific literature (10; 2.7%). Only 72 (19.2%) participants consulted healthcare providers before initiating alternative therapies.

The community appeared divided on recommending alternative treatments, with 183 (50.6%) in favour and 179 (49.4%) against.

### 3.4 Associations discovered in the studied sample

Analysis of factors associated with mental health diagnoses and help-seeking behaviour revealed several significant patterns (Tables 4–7). Female respondents demonstrated approximately twice the prevalence compared to males (52.94% vs. 26.76%, *p* < 0.001).

**Table 4:** Demographic and behavioural factors associated with psychiatric diagnosis or help-seeking.

Factor	Category	Psychiatric diagnosis/help-seeking rate (%)	Comparison	*p*-value
Sex and psychiatric diagnosis	Female (*n* = 340)	52.94	vs. Male	**<0.001**
	Male (*n* = 142)	26.76	-	-
	Other (*n* = 11)	100	number to small for comparison	
Smoking status and psychiatric help-seeking	Smokers (*n* = 114)	53.51	vs. Non-smokers	**<0.001**
	Non-smokers (*n* = 378)	42.86	-	-
Field of study and mental health diagnosis	Engineering (*n* = 111)	57.66	vs. Social sciences	**<0.001**
	Humanities (*n* = 42)	83.33	vs. Engineering	**<0.001**
	Healthcare (*n* = 246)	69.51	vs. Social sciences	**<0.001**
	Social sciences (*n* = 74)	94.59	-	-

The data represent associations between demographic/behavioural characteristics and psychiatric diagnosis or help-seeking behaviour. Female respondents showed approximately twice the diagnosis rate compared to males. All intersex respondents (100%) reported a psychiatric diagnosis, however this group (*n* = 11) was too small to draw significant conclusions. Smoking status was significantly associated with help-seeking behaviour. Field of study comparisons indicate relative diagnostic prevalence patterns. Bold values indicate statistical significance (*p*<0.05).

**Table 5:** Demographic and behavioural factors associated with psychiatric diagnosis or help-seeking.

Category/substance	Professional support seekers (%) (*n* = 354)	No professional support (%) (*n* = 136)	*p*-value
Marijuana	34.46	21.32	**0.005**
Probiotics	8.76	2.21	**0.011**
Vitamins	26.27	11.76	**0.001**

The data represent associations between the percentage of respondents seeking professional psychological support in subgroups divided based on the usage of particular substances. Bold values indicate statistical significance (*p*<0.05).

**Table 6:** Demographic and behavioural factors associated with psychiatric diagnosis or help-seeking.

Herbal/substance use by sex	Female (%) (*n* = 340)	Male (%) (*n* = 142)	*p*-value
Chamomile	25.29	10.56	**<0.001**
Lavender	13.82	4.23	**0.002**
Lemon balm	61.47	32.39	**<0.001**
*Amanita muscaria*	0.59	4.23	**0.004**
Probiotics	7.65	1.41	**0.008**
Vitamins	23.82	10.56	**0.001**

Substance use comparison between male and female respondents. No statistically significant differences were observed for other substances examined. Bold values indicate statistical significance (*p*<0.05).

**Table 7:** Depression severity (PHQ-10 scores) and substance use patterns.

Substance	Mean PHQ-10 (95% CI)		*p*-value
	Users of the particular substance	Non-users	
Marijuana	15.54 [14.39–16.69]	13.49 [12.77–14.22]	**0.004**
Ashwagandha	19.22 [17.72–20.73]	13.4 [12.76–14.05]	**<0.001**
St. John’s wort	17.97 [15.67–20.27]	13.76 [13.13–14.40]	**<0.001**
Psychedelics	15.29 [13.34–17.24]	Non-users: 13.93 [13.28–14.58]	0.213

Mean PHQ-10 scores in substance users and control comparison groups with a 95% confidence interval (95% CI). Bold values indicate statistical significance (*p*<0.05).

A highly significant association was observed between smoking status and psychiatric help-seeking behaviour in the student sample - More students who reported smoking had sought psychiatric help, compared to non-smoking students. Field of study also significantly influenced diagnosis rates, with engineering students showing lower prevalence compared to social sciences and humanities students (Table 4.).

Professional help-seeking patterns were assessed in subgroups divided based on the usage of particular substances. No significant difference was observed in general herbal supplement use between those who had and had not sought professional mental health support (81.17% vs. 74.56%; *p* = 0.06). However, significant differences emerged in specific substance usage—the significant relationships were depicted in Table 5.

Significant sex differences were observed in the use of herbal supplements and other substances (Table 6). Female participants demonstrated significantly higher usage rates of herbal remedies overall.

Analysis of PHQ-10 scores revealed differences between users and non-users of certain substances, which were depicted in Table 7.

The relationship between yoga practice and substance use revealed selective associations. Yoga practitioners reported significantly higher marijuana usage (35.23%) compared to non-practitioners (22.33%, *p* = 0.002). However, no significant difference was observed in psychedelic use between yoga practitioners (10.36%) and non-practitioners (8.33%, *p* = 0.445).

Students with ADHD demonstrated different substance use patterns compared to those with other diagnosed disorders. ADHD-diagnosed participants reported significantly higher marijuana use (45.83%) compared to participants with other disorders (29.5%, *p* = 0.02). However, psychedelic use showed no significant difference between ADHD-diagnosed individuals (12.5%) and those with other conditions (8.78%, *p* = 0.396).

Analysis of barriers to conventional psychiatric/psychological care revealed that the primary deterrents among students using alternative methods were cost (58.9%), accessibility (25.8%), and lack of trust in therapy effectiveness (21.5%).

Attitudes towards novel psychiatric therapies with ketamine and psilocybin were examined among subgroups of marijuana, psychedelics, yoga, and meditation users. The results were depicted in Table 8.

**Table 8:** Willingness to undergo ketamine/psilocybin therapy in subgroups.

Willingness to undergo novel therapies		Users (%)	Non-users (%)	*p*-value
Marijuana users	Ketamine therapy	34.81 (*n* = 47/135)	9.8 (*n* = 35/357)	**<0.001**
	Psilocybin therapy	40.74 (*n* = 55/135)	11.76 (*n* = 42/357)	**<0.001**
Psychedelic users	Ketamine therapy	55.56 (*n* = 25/45)	12.75 (*n* = 57/447)	**<0.001**
	Psilocybin therapy	66.67 (*n* = 30/45)	14.99 (*n* = 67/447)	**<0.001**
Yoga practitioners	Ketamine therapy	20.21 (*n* = 39/193)	14.38 (*n* = 43/299)	**0.09**
	Psilocybin therapy	24.87 (*n* = 48/193)	16.39 (*n* = 49/299)	**0.021**
Meditation practitioners	Ketamine therapy	19.51 (*n* = 56/287)	12.68 (*n* = 26/205)	**0.045**
	Psilocybin therapy	23 (*n* = 66/287)	15.12 (*n* = 31/205)	**0.03**

Percentage of substance users or yoga/meditation practitioners accepting ketamine and psilocybin therapy compared to the corresponding percentage of acceptance among non-users/non-practitioners, depicted along with the p-value for comparison. These responses reflect participants’ personal readiness to receive treatment if professionally recommended, rather than their beliefs about therapeutic efficacy or general theoretical approval of these modalities. Bold values indicate statistical significance (*p*<0.05).

Some of the analysed data revealed no expected associations. No significant association was found between marital status or academic disciplines and use of alternative medicine methods and self-administered substances, suggesting comparable utilisation of CAM regardless of study field and marital status. There is also no association between employment status and a pattern of seeking help from mental health professionals. Assessment of psychiatric care satisfaction revealed no significant differences between substance users and non-users, suggesting that neither marijuana nor psychedelics use has an impact on perceived quality of psychiatric care.

## 4 Discussion

### 4.1 Factors affecting propensity to use alternative treatment methods

Our study shows that female students in Wrocław are significantly more likely to report having received a psychiatric diagnosis than males. These findings are consistent with those observed for the general population (37). However, our study also revealed significant differences between male and female students in the utilisation of CAM methods for coping with mental health issues, particularly in the use of herbal remedies. Female participants were more likely than males to use chamomile, lavender, and lemon balm. This difference aligns with existing literature suggesting that women are more inclined to seek out CAM for mental health support. For example, in one study, women with migraines or severe headaches were found to be significantly more likely than men to use CAM approaches, including herbal supplements, and this use was associated with lower levels of moderate mental distress among women—but not among men (38). Similarly, a secondary analysis of the 2012 US National Health Interview Survey showed that female CAM users were more likely to report using such therapies for general well-being and mental health reasons, compared to male users (39). Moreover, differences in CAM use between sexes are not restricted to mental health conditions. As demonstrated in one study, women with multiple chronic physical conditions were more likely than men to use various CAM modalities, including herbal therapies, suggesting a broader trend of higher CAM engagement among women across different health contexts (40).

In contrast, male students reported significantly higher usage of *A. muscaria* compared to female participants. Similar patterns were observed in a study by Ordak et al., which analysed consumption patterns of *A. muscaria* among internet users (41). The researchers found that men were significantly more likely to consume dried *A. muscaria* preparations, while women more often used tinctures. Additionally, male users more frequently reported using *A. muscaria* to self-manage symptoms of stress, depression, and insomnia, suggesting a tendency towards autonomous and potentially riskier coping strategies involving use of psychoactive substances. The study also noted that adverse effects such as nausea, vomiting, abdominal pain, and drowsiness were reported more frequently by men, which may further support the notion of more intense or unregulated use patterns. A study by Hartwig et al., revealed that declared reasons for use of *A. muscaria* include supposed reduction in use of other substances (alcohol, benzodiazepines, marijuana etc.), alleviation of anxiety, depression and other psychiatric disorders (42). Therefore, there is a possibility of harmful pharmacological interactions with other substances or prescribed drugs, as well as the unpredictable influence of *A. muscaria* on mental state. Use of *A. muscaria* can also lead to acute intoxication with symptoms such as respiratory failure and coma (43, 44). In our opinion, those factors combined create an issue that should be addressed by further issue, especially concerning male students.

Despite the observed differences in psychiatric diagnosis rates across academic disciplines, no significant differences were identified in the frequency of utilising alternative treatment methods among students from various fields of study. This observation is in line with findings from a study conducted among university students in Atlanta, New Delhi, and Newcastle upon Tyne (45). Similarly, the relationship status of participants has no impact on their therapeutic choices. Both individuals in romantic relationships and single individuals demonstrated similar tendencies to engage in alternative forms of treatment. Widespread use of CAM among students, independent of the mentioned factors, suggests that a shift towards integrative approaches to health is a broader cultural trend. This may be caused by the common occurrence of CAM in media, which is reflected in our study results - for 78.5% of students media were a source of information about alternative therapies. Moreover, 47.0% of respondents spent more than 3 h daily using social media. The popularity of CAM in the media may be stimulated by paid advertising, especially in the case of legal products, such as herbal supplements. Even though our analysis revealed no association between social media and CAM usage. More comprehensive research is needed to assess cultural factors affecting CAM popularity among students.

Another factor influencing the popularity of CAM among Wrocław students is the lack of access to conventional, professional help. Respondents in our study pointed out that cost is the main obstacle (80.7%). The population of university students is particularly vulnerable to high costs of private psychiatric and psychological care - as our study shows, only 45.8% of students were employed during their education. Also availability of public mental health care was a limiting factor for 35.7% of respondents. As for Wrocław, waiting time for psychiatric consultation ranges from 21 days to over 3 years, however, in most institutions, patients have to wait 2–6 months (46). Obstacles revealed by our study also include psychological factors: fear of stigma, lack of physicians’ empathy and fear of judgment. These prejudices can be aggravated by a current mental health disorder in patients with anxiety and negative beliefs, but do not necessarily lack rationality: a study on undergraduate medical students from 65 countries showed widespread occurrence of stigmatisation of psychiatry and psychiatric patients (47). However, the stigma associated with mental health and psychiatric care is a complex, multidimensional phenomenon that differs significantly across ethnic and cultural contexts (48). In a study by Newberry et al., the two most common barriers preventing patients from accessing mental healthcare were concerns about treatments available (65%) and financial cost (62.7%) (49). “Being seen as weak” was an additional reported barrier, although not the prime one. Our study highlights that stigmatisation is still a significant issue that should be considered by public health specialists in some populations. More research is needed to analyse cultural and societal stigma in the context of academia, particularly in Poland.

### 4.2 Usage of marijuana and psychedelics and its two-way relationship with mental health

In our study, marijuana users demonstrated significantly more intense depressive symptoms compared to non-users. A similar observation has been made in other studies concerning the population of university students (50–52). Two models have been postulated to explain this association. The first is known as the precipitation model (53), whereby drug use may trigger psychopathology via neuroadaptation in brain reward pathways that can lead to subsequent development of psychiatric disorders. Individuals who routinely use marijuana have significantly altered brain structures and functions, including cognition and mood regulation, which may potentially result in long-lasting amotivational syndrome (54). One longitudinal study found that cannabis use was associated with an increased number of depressive symptoms at follow-up, including anhedonia, changes in body weight, sleep disorders, and psychomotor problems (55). A second hypothesis explaining the aforementioned association is the self-medication model (56), which postulates that psychiatric disorders may cause an increase in drug use due to their ability to alleviate some of the mental health symptoms. Indeed, individuals with clinical or subthreshold depression may find acute relief of symptoms as cannabis intoxication can induce positive effects on mood (e.g., euphoria) and relaxation (57). Interestingly, research on the effects of acute intoxication in adulthood shows that cannabis reduces amygdala response to negative faces, potentially reflecting an anxiolytic effect that can reduce depressive symptoms (58).

In our study, marijuana consumption was higher among those who had received professional support compared to those who had not. Apart from the above-mentioned association between depressive symptoms and cannabis use, it might also be partially explained by the fact that Poland presents a liberal approach to medical cannabis prescriptions. The Ministry of Health requires no additional training or certificates for prescribing herbal cannabis. Furthermore, the decision to initiate treatment and the responsibility for choosing proper indication and dosing relies solely on the prescribing physician because no official guidelines define these. As a result, some physicians may prescribe medications for psychiatric indications (59). Although there is weak evidence for cannabinoids’ beneficial effects in anxiety and mood disorders, their use in psychiatric disorders remains largely unexplored due to a lack of valid, reliable, empirical evidence (60, 61). This issue should be further investigated and addressed by public health policies to avoid the risk of cannabinoids’ negative impact on psychiatric patients. We suggest that physicians treating students should always inquire about their marijuana consumption and provide them with information about marijuana’s adverse effects, e.g., in the form of a psychoeducative leaflet.

### 4.3 Herbal preparations as complementary treatment—possibilities and perils

Although our study showed that the majority of students were satisfied with professional mental health care (33.6% reported the highest satisfaction and 30.5% very high satisfaction), as well as psychotropic drugs (29.3 and 33.9%, respectively), patients who use alternative methods for treating mental problems rarely consult them with specialists such as psychiatrists or psychologists (19.2%). Students either take both prescribed drugs and self-administered substances at the same time, or they choose alternative therapy instead of consulting a psychiatrist and conventional therapy. In the first case, such behaviour can result in potentially harmful drug-substance interactions.

One of the most notable examples is st. John’s wort (*Hypericum perforatum*), which contains the active substance hyperforin. It can induce cytochrome P450 enzymes, mainly CYP3A4 and change the pharmacokinetics of many drugs, including benzodiazepines and carbamazepine (62). In the Polish population, cases of clinically significant interactions were also noted between st. John’s wort and duloxetine, haloperidol, quetiapine, clorazepate, clonazepam (63). In conjunction with other serotonergic drugs it can cause even fatal serotonin syndrome (64). Furthermore, hyperforin alone can have side effects, such as gastrointestinal symptoms, dizziness, confusion, tiredness, sedation and photosensitivity (65, 66). Our study showed that the prevalence of st. John’s wort intake among students is high (11.6%), potentially making it a significant problem for public health. However, as some evidence shows the efficiency of st. John’s wort in the treatment of mild to moderate depression (67), its usage under a psychiatrist’s supervision can be taken into consideration, especially in patients who are reluctant to conventional treatment.

Another herbal medicine, ashwagandha (*Withania somnifera*), was used by 24.8% of participants. It is considered potentially beneficial for stress and anxiety (68, 69) or even schizophrenia (70). Even though the evidence suggests that ashwagandha can be used in the treatment of anxiety, its efficacy is still unsatisfactorily assessed (71). Our research showed that ashwagandha users also exhibited significantly higher PHQ-10 scores compared to non-users. This may be interpreted through the lens of the self-medication model: students with more severe mental health problems more often use such substances to alleviate their symptoms. As previously said, some studies have reported beneficial effects of ashwagandha supplementation in depressive symptoms (72, 73), as well as anti-stress and anti-anxiety effects in both animal models and clinical research (74, 75). It is well established that stress can lead to both functional and structural brain changes and is a known contributor to depression (76). As such, the antidepressant effects of ashwagandha may stem from its ability to regulate the hypothalamic–pituitary–adrenal (HPA) axis (74).

As for safety, adverse events were identified in ashwagandha users, notably liver toxicity, probably through DNA damage (77). It is also suggested that ashwagandha root extract may be a CYP3A4 inducer or CYP2B6 inhibitor, which can lead to pharmacological interactions (78). These two examples show that self-administered therapy with herbal medicine, especially in combination with conventional drugs, can lead to harmful effects. Our study suggests that incidence may be high, as many students use such substances. However, our study shows that the most common herb among students in Wrocław was lemon balm (*Melissa officinalis*)(used by 53.0% respondents), which is considered substantially safe, with very rare adverse events (79). Some evidence even suggests that it may be effective in anxiety and depressive symptoms, although studies are highly heterogeneous and further research is needed (80). Similarly, yellow chamomile (*Matricaria chamomilla*; 21.2%) is rather safe and can help improve the quality of sleep or in generalised anxiety disorder (GAD) (81). In conclusion, some of the commonly used herbs can be helpful as complementary therapy in less severe disorders, but specialist surveillance is recommended, especially in the case of ashwagandha and st. John’s wort. Therefore, we suggest active screening for the use of non-prescribed substances during psychiatric consultation, especially in the population of university students.

### 4.4 A link between modern and alternative therapies: ketamine, psychedelics, yoga, and meditation

The renaissance of research in the field of psychedelic therapy brought public attention to therapeutic usage of lysergic acid diethylamide (LSD), psilocybin, ketamine etc. in psychiatric disorders. As long as there is evidence of efficacy of particular psychedelic therapies (82, 83), concerns and problems in methodology affect the reliability of studies involving administration of psychedelics. In result, in most countries, including Poland, psychedelics aren’t registered in therapy other than experimental, apart from esketamine, registered for treatment-resistant depression (84). As this subject sparks controversy among specialists, the attitude of patients towards such therapy can be unenthusiastic. Our study showed that fewer students would agree to both ketamine and psychedelic therapy (19.0 and 22.5% respectively) than to classic psychiatric medications (75.9%). What’s interesting, acceptance for scientifically proven and relatively safe electroconvulsive therapy was even lower (18.6%), suggesting that other factors than hard evidence may have an impact on public opinion, such as depictions in culture or associated stereotypes. In the case of psychedelics and ketamine, our study showed that their acceptance was significantly higher among students who already used marijuana and psychedelics, as well as in meditation practitioners and yoga practitioners, but in this case only for psychedelics, not ketamine. These findings are supported by literature showing a link between meditation practice and classic psychedelics usage, which both lead to disruption of self-consciousness and ego dissolution (85, 86). Yoga and meditation are also often associated with open-mindedness. As Turiano et al. noted in their study, higher levels of openness, among few other personality traits, predicted longitudinal substance use (87). Health professionals should remain aware of the potential vulnerability of yoga and meditation practitioners to drug use (88). This appears to be consistent with our results.

Although individuals who engage in yoga and meditation may exhibit a higher likelihood of substance use, the consistent positive associations between these practices and a range of health-promoting behaviours justify their inclusion in preventive medicine and integrative healthcare approaches (88). Its positive influence on mental well-being has been extensively touched upon in numerous studies (89–92). Moreover, our study revealed that physical activity is the predominant method of self-initiated mood improvement strategies among students. The role of exercise in mental health improvement was previously determined, showing its positive impact on psychological well-being (93, 94). Physical activity can also be utilised to treat concomitant diseases and metabolic side effects of psychiatric medications. As the positive influence of physical activity, yoga, and meditation on mental health can be clearly noticed, and as, according to the results of our study, it is widely accepted by patients, those CAM methods should be considered in therapeutic strategies by mental health specialists.

### 4.5 Limitations

Even though the authors maintained the regulations of ICC/ESOMAR International Code on Market, Opinion and Social Research and Data Analytics, this study has several limitations that should be acknowledged. First, the use of a convenience sampling method and voluntary participation may have introduced self-selection bias, as students with a particular interest in mental health or alternative therapies may have been more likely to respond. The percentage of respondents with a previous psychiatric diagnosis in the sample was 46.5%, which seems to be high, however, studies on larger groups show the prevalence of mental health problems among students is even up to 60% (95). Second, the cross-sectional design does not allow for causal inferences regarding the relationships between mental health status and the use of alternative treatments. Third, all data were self-reported, which may be subject to recall bias or social desirability bias, particularly in responses related to sensitive topics such as psychiatric diagnoses or use of psychoactive substances. Additionally, although efforts were made to reach a diverse student population, the sample may not be fully representative of all students across Wrocław universities. Lastly, a modified version of the PHQ-9 with an extended timeframe (12 months instead of 2 weeks) was used. While this modification allowed for capturing longer-term mood disturbances relevant to the research subject, it represents a deviation from the validated instrument. The modified version was not independently validated, and the standard PHQ-9 cut-off scores may not be directly applicable to a 12-month recall period. The tool was used for screening purposes to identify the presence of depressive symptoms and mood disturbances, not for clinical diagnosis. Future studies should consider using both short-term and long-term assessment tools or conduct validation studies for extended timeframe versions of standardised instruments.

## 5 Conclusion and future directions

Our study shows that the prevalence of alternative and self-administered methods of treatment of mental disorders among students in Wrocław is high, with the most popular being herbs, marijuana, vitamins, psychedelics, as well as physical activity, meditation, and yoga. Popularity of CAM differs between sexes and psychiatric diagnoses, but is not affected by study field, mode, and relationship status. More students practising yoga or meditation use substances such as marijuana and have greater acceptance for modern psychiatric therapies, such as ketamine treatment. The reasons for alternative methods may be obstacles in accessing professional care, as well as cultural influences and psychological mechanisms. The main risks associated with CAM are a lack of specialists’ surveillance over self-applied treatment, the possibility of drug interactions and adverse events, or substance use disorder.

Mental health professionals working with the population of students should be aware of our findings. Firstly, we suggest that they should always ask if a patient is using CAM and assess their safety, especially in combination with prescribed psychotropic drugs. Moreover, better education of the students’ community is needed to increase acceptance for classic therapies, especially ECT. Secondly, psychiatric and psychological care must be more accessible for students to facilitate early diagnosis and use of reliable, professional therapies instead of self-administration of substances such as marijuana. Finally, some of the CAM methods, e.g., physical activity, meditation, breathing techniques, and yoga, can potentially be a helpful addition in the therapeutic process, and future research should examine their efficacy. Guidelines and public health strategies should be developed to address risks coming from the high prevalence of CAM, especially marijuana among students. As the results of this study are limited to the Wrocław students’ population, surveys in different cities, countries, age groups, and cultural contexts should be conducted for a more thorough understanding of the phenomenon.

## Data Availability

The raw data supporting the conclusions of this article will be made available by the authors, without undue reservation.
