# Supplementary material for: Self-administered complementary and alternative methods of treating mental disorders among students in Wrocław: a cross-sectional study
Source: Front Public Health. 2026 Jan 8;13:1734137. doi: 10.3389/fpubh.2025.1734137 (PMC12823540; doi:10.3389/fpubh.2025.1734137)
Supplement: Supplementary file 1 [file Supplementary_file_1.pdf]

Supplementary material to article “Self-administered complementary and alternative methods of treating mental disorders among students in Wrocław: a cross-sectional study”

1. Original questionnaire text

Drodzy Studenci,

Prosimy Was o udział w badaniu prowadzonym w ramach akcji "Psychiatria też dla ludzi". Naszym celem jest zbadanie zjawisk związanych z unikaniem szukania pomocy psychiatrycznej i stosowaniem alternatywnych metod radzenia sobie z zaburzeniami nastroju wśród studentów wrocławskich uczelni.

Ankieta jest w pełni anonimowa, w każdej chwili można zrezygnować z jej wypełniania. Wypełniając i przesyłając tę anonimową ankietę, oświadczasz, że zapoznałeś się z podanymi informacjami, rozumiesz cel badania i dobrowolnie wyrażasz zgodę na udział.

Całość powinna zająć nie więcej niż 10 minut.

Z góry dziękujemy za poświęcony czas.

Studenci z SKN Psychiatrii

Uniwersytet Medyczny we Wrocławiu

Q1: Ile ma Pani/Pan lat?

Tak

Nie

Q2: Płeć:

Kobieta

Mężczyzna

Inna

Q3: Jestem studentem studiów:

dziennych

zaocznych

wieczorowych

Q4: Studiuję kierunek (nazwa kierunku):

Q5: Czy oprócz nauki Pani/Pan pracuje?

Nie

Tak

Q6: Jaki jest Pana/Pani stan cywilny?

Małżeństwo

Związek nieformalny

Singiel/singielka

Q7: Ile średnio korzysta Pan/Pani z mediów społecznościowych (Facebook, Instagram, TikTok itd.)?

mniej niż 3 godziny dziennie

więcej niż 3 godziny dziennie

kilka razy w tygodniu

raz w tygodniu lub rzadziej

nie korzystam

Q8: Czy pali Pan/Pani papierosy (lub inne wyroby tytoniowe)?

Tak

Nie

Q9: Czy miał Pan/Pani kiedykolwiek zdiagnozowane przez psychiatrę zaburzenia psychiczne?

Tak

Nie

Q10: Jeśli tak, to które?
Depresja

Schizofrenia

Zaburzenia lękowe

Choroba afektywna dwubiegunowa

Zaburzenia odżywiania

Zaburzenia obsesyjno-kompulsywne (OCD)

ADHD

Inne:

Q11: Czy kiedykolwiek był Pan/Pani u psychiatry lub w szpitalu psychiatrycznym?

Tak

Nie

Q12: Jak bardzo jest Pan/Pani zadowolona z opieki psychiatry?

Nie jestem zadowolony/a 1 2 3 4 5 Bardzo zadowolony/a

Q13: Co Pana/Panią zniechęca do korzystania z profesjonalnej opieki psychiatrycznej?

Koszt

Dostępność

Nie wierzę w skuteczność leczenia

Obawiam się negatywnej oceny otoczenia w związku z terapią

Brak empatii ze strony lekarza

Obawiam się negatywnych skutków leków

Nie zgadzam się z diagnozą

Inne:

Q14: Czy kiedykolwiek korzystał Pan/Pani z pomocy psychologa lub psychoterapeuty?

Tak, obecnie

Tak, w przeszłości

Nie

Q15: Jak bardzo jest Pan/Pani zadowolony z opieki psychologa lub psychoterapeuty?

Nie jestem zadowolony/a 1 2 3 4 5 Bardzo zadowolony/a

Q16: Co Pana/Panią zniechęca do korzystania z pomocy psychologa/psychoterapeuty?

Koszt

Dostępność

Nie wierzę w skuteczność leczenia

Nie mam czasu na regularne wizyty

Obawiam się negatywnej oceny otoczenia w związku z terapią

Brak zaufania do psychologa/psychoterapeuty

Nie zgadzam się z diagnozą

Inne:

Q17: Czy kiedykolwiek stosował/a Pan/Pani leki psychotropowe (np. na depresję) przepisane przez lekarza?

Nie

Tak, w przeszłości

Tak, obecnie

Q18: Jak bardzo jest Pan/Pani zadowolony z leków przepisanych przez psychiatrę?

Nie jestem zadowolony/a 1 2 3 4 5 Bardzo zadowolony/a

Q19: Na które z metod leczenia, zaproponowanych przez lekarza psychiatrę, zgodziłby się Pan/Pani?

Leki psychotropowe (np. przeciwdepresyjne)

Leki ziołowe (np. ekstrakt z dziurawca)

Psychoterapia

Elektrowstrząsy

Przezczaszkowa stymulacja magnetyczna

Terapia ketaminą

Terapia psylocybiną

Terapia EMDR (eye movement desensitization and reprocessing)

Żadną z powyższych

Hipnoterapia

Q20: Czy stosował Pan/Pani kiedykolwiek któryś z poniższych sposobów w celu poprawy obniżonego nastroju?

Żaden z poniższych

Akupunktura

Dieta poprawiająca nastrój

Joga

Medytacja, techniki relaksacyjne

Aromaterapia

Oddychanie holotropowe

Wysiłek fizyczny

Hipnoza

Modlitwa

Inne:

Q21: Jak bardzo jest Pan/Pani zadowolona z powyższych sposobów?

Nie jestem zadowolony/a 1 2 3 4 5 Bardzo zadowolony/a

Q22: Czy w celu poprawy swojego samopoczucia lub zdrowia psychicznego sięgał Pan/Pani po preparaty ziołowe zawierające któreś z podanych roślin?

Dziurawiec zwyczajny

Ogórecznik właściwy

Rumianek żółty

Lawenda wąskolistna

Melisa lekarska

Kozłek lekarski

Kwiat gorzkiej pomarańczy

Szafran

Kminek

Pieprz metystynowy

Miłorząb (Gingko biloba)

Rozchodnik różowy

Bazylia święta

Ashwagandha (witania ospała)

Żadna z powyższych

Inne:

Q23: Czy stosował Pan/Pani którąś z poniższych substancji w celu poprawy samopoczucia psychicznego, nastroju?

Żaden z poniższych

Muchomor czerwony (Amanita muscaria)

Psychodeliki (LSD, DMT, meskalina, grzyby halucynogenne)

Ketamina

Marihuana, CBD

Probiotyk (psychobiotyk)

Preparaty witaminowe

Leki homeopatyczne

Inne:

Q24: Jaki wpływ na Pana/Pani samopoczucie psychiczne miało Pana/Pani zdaniem stosowanie powyższych substancji?

Bardzo negatywny 1 2 3 4 5 Bardzo pozytywny

Q25: Skąd czerpałeś informacje dotyczące alternatywnych metod terapii?

Przyjaciele, znajomi i członkowie rodziny

Lekarze

Media (internet, telewizja, książki i czasopisma)

Inne:

Q26: Czy konsultowałeś stosowanie alternatywnych metod terapii z lekarzem?

Tak

Nie

Q27: Czy poleciłbyś stosowanie alternatywnych metod terapii innym?

Tak

Nie

Q28: Jak często w ciągu ostatnich 12 miesięcy dokuczały Panu/Pani następujące problemy? Proszę zaznaczyć odpowiedź.

Wcale nie dokuczały

Kilka dni

Więcej niż połowę dni

Niemal codziennie

Niewielkie zainteresowanie lub odczuwanie przyjemności

Uczucie smutku, przygnębienia lub beznadziejności

Kłopoty z zaśnięciem lub przerywany sen, albo zbyt długi

Uczucie zmęczenia lub brak energii

Brak apetytu lub przejadanie się

Poczucie niezadowolenia z siebie — lub uczucie, że jest się do niczego, albo że zawiódł/zawiodła Pan/Pani siebie lub rodzinę

Problemy ze skupieniem się na przykład przy czytaniu

Poruszanie się lub mówienie tak wolno, że inni mogliby to zauważyć? Albo wręcz przeciwnie — niemożność usiedzenia w miejscu lub poddenerwowanie powodujące ruchliwość znacznie większą niż zwykle

Myśli, że lepiej byłoby umrzeć, albo chęć zrobienia sobie jakiejś krzywdy

Niewielkie zainteresowanie lub odczuwanie przyjemności

Uczucie smutku, przygnębienia lub beznadziejności

Kłopoty z zaśnięciem lub przerywany sen, albo zbyt długi

Uczucie zmęczenia lub brak energii

Brak apetytu lub przejadanie się

Poczucie niezadowolenia z siebie — lub uczucie, że jest się do niczego, albo że zawiódł/zawiodła Pan/Pani siebie lub rodzinę

Problemy ze skupieniem się na przykład przy czytaniu

Poruszanie się lub mówienie tak wolno, że inni mogliby to zauważyć? Albo wręcz przeciwnie — niemożność usiedzenia w miejscu lub poddenerwowanie powodujące ruchliwość znacznie większą niż zwykle

Myśli, że lepiej byłoby umrzeć, albo chęć zrobienia sobie jakiejś krzywdy

Q29: Jeżeli zaznaczył/-a Pan/Pani którekolwiek z problemów, jak bardzo utrudniły one Panu/Pani wykonywanie pracy, zajmowanie się domem lub relacje z innymi ludźmi?

Nie zaznaczyłem żadnego z problemów

W ogóle nie utrudniły

Trochę utrudniły

Bardzo utrudniły

Niezmiernie utrudniły

Q30: Ile różnych leków przeciwdepresyjnych na receptę obecnie zażywasz lub przyjmowałeś w przeszłości (łącznie)?

0

1

2-4

≥5

2. CHERRIES checklist.

CHERRIES Item	Addressed in the Present Study
**1. Design**	Cross-sectional online survey; target population defined as students of Wrocław universities.
**2. IRB Approval**	Not necessary
**3. Informed Consent**	Participants were presented with an introductory statement at the beginning of the Google Forms questionnaire, explaining the purpose of the study, that responses were anonymous, proceeding to the survey indicated consent (“implied consent”).
**4. Data Protection**	Responses stored in digital environment accessible only to research team.
**5. Development and Pre-testing**	The research team generated an initial pool of items based on a review of existing literature on student mental health and CAM, technical functionality was evaluated on multiple devices
**6. Recruitment process and description of the sample having access to the questionnaire**	Survey link publicly distributed via social media, multi-channel recruitment via Facebook, printed posters, and in-person lectures.
**7. Survey administration**	Web-based survey administered via Google Forms, voluntary participation, conducted from April to December 2024, participants could review or change answers before submission
**8. Response rates**	View rate, participation rate and completion rate were

	not reported
**9. Preventing multiple entries from the same participant**	No methods were used to identify multiple entries from the same participant
**10. Analysis**	Five incomplete responses were excluded, only responses from April to December 2024 were analysed

3. Original graphic materials for survey repondents recruitment.

Fig. 1. Online leaflet used for recruitment to the study in the Internet. Created by Marta Błażejewska.

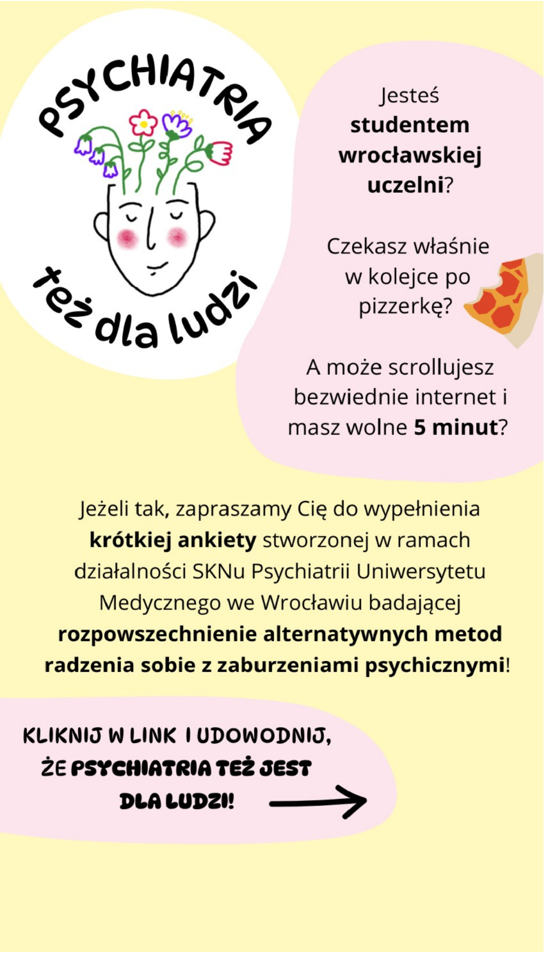

Fig. 2. Leaflet used for recruitment to the study. Created by Marta Błażejewska.

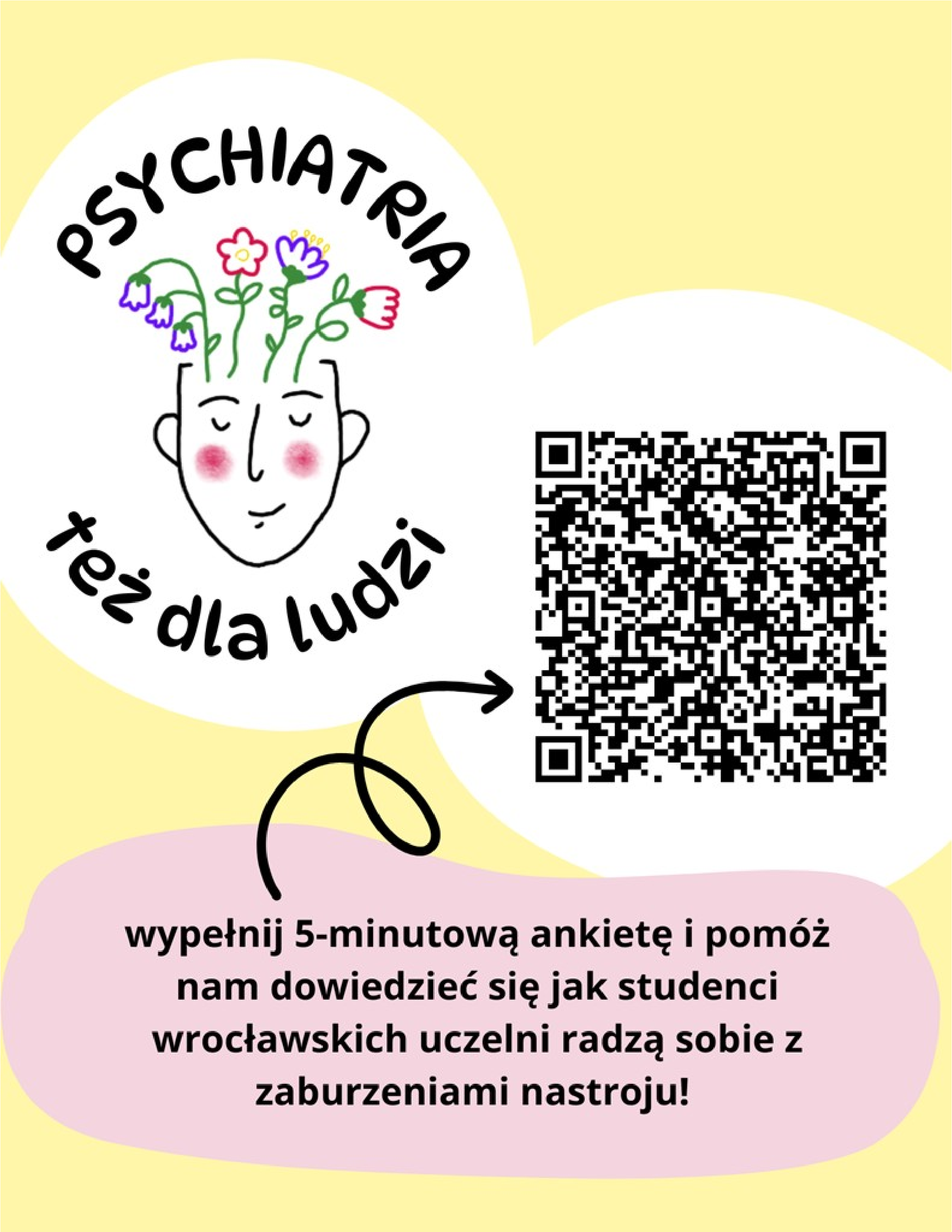

Fig. 3. Poster used for recruitment in the study. Created by Marta Błażejwska.

PSYCHIATRIA też dla ludzi

Jesteś **studentem wrocławskiej uczelni**?

Czekasz właśnie w kolejce po pizzerkę?

A może scrollujesz bezwiednie internet i masz wolne **5 minut**?

Jeżeli tak, zapraszamy Cię do wypełnienia **krótkiej ankiety** stworzonej w ramach działalności SKNu Psychiatrii Uniwersytetu Medycznego we Wrocławiu badającej **rozpowszechnienie alternatywnych metod radzenia sobie z zaburzeniami psychicznymi**!

↓ KLIKNIJ W LINK W OPISIE I UDOWODNIJ, ŻE **PSYCHIATRIA TEŻ JEST DLA LUDZI!**
